# PRD-Class Homeobox Genes in Bovine Early Embryos: Function, Evolution, and Overlapping Roles

**DOI:** 10.1093/molbev/msac098

**Published:** 2022-05-05

**Authors:** Thomas D. Lewin, Ali A. Fouladi-Nashta, Peter W.H. Holland

**Affiliations:** Department of Zoology, University of Oxford, 11a Mansfield Road, Oxford OX1 3SZ, UK; Comparative Biomedical Sciences Department, Royal Veterinary College, Hawkshead Campus, North Mymms, Hatfield, Hertfordshire AL9 7TA, UK; Department of Zoology, University of Oxford, 11a Mansfield Road, Oxford OX1 3SZ, UK

**Keywords:** homeodomain, blastocyst, Crx, genetic redundancy, protein evolution, gene

## Abstract

Eutherian Totipotent Cell Homeobox (ETCHbox) genes are mammalian-specific PRD-class homeobox genes with conserved expression in the preimplantation embryo but fast-evolving and highly divergent sequences. Here, we exploit an ectopic expression approach to examine the role of bovine ETCHbox genes and show that ARGFX and LEUTX homeodomain proteins upregulate genes normally expressed in the blastocyst; the identities of the regulated genes suggest that, in vivo, the ETCHbox genes play a role in coordinating the physical formation of the blastocyst structure. Both genes also downregulate genes expressed earlier during development and genes associated with an undifferentiated cell state, possibly via the JAK/STAT pathway. We find evidence that bovine *ARGFX* and *LEUTX* have overlapping functions, in contrast to their antagonistic roles in humans. Finally, we characterize a mutant bovine *ARGFX* allele which eliminates the homeodomain and show that homozygous mutants are viable. These data support the hypothesis of functional overlap between ETCHbox genes within a species, roles for ETCHbox genes in blastocyst formation and the change of their functions over evolutionary time.

## Introduction

Eutherian Totipotent Cell Homeobox (ETCHbox) genes are a group of highly variable PRD-class homeobox genes specific to mammals. In humans, ETCHbox genes are expressed almost exclusively in the preimplantation embryo between the 4-cell stage and blastocyst, and this temporal expression pattern is broadly conserved in cattle (Madissoon et al. 2016; Maeso et al. 2016). The genes are thought to be involved in key transitions during embryonic development, acting as a vital component of regulatory networks active at the 8-cell stage and in the morula (Jouhilahti et al. 2016; Madissoon et al. 2016; Maeso et al. 2016; Mori et al. 2018; Royall et al. 2018; Taubenschmid-Stowers et al. 2022; Mazid et al. 2022).

**Fig. 1.**
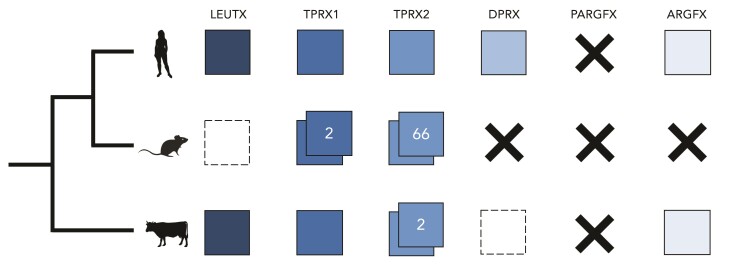
ETCHbox gene repertoires of human, mouse, and cattle. Colored squares indicate the presence of a functional gene. White squares with dashed borders represent pseudogenes, and a black X represents complete gene loss. Double squares indicate duplication; numbers show the quantity of duplicates of a given gene present in the genome.

The six ancestral ETCHbox genes (*ARGFX*, *DPRX*, *LEUTX*, *PARGFX*, *TPRX1*, and *TPRX2*) duplicated from the Otx-family gene *CRX* on the stem lineage leading to eutherians, and the ancestral eutherian ETCHbox repertoire likely consisted of one copy of each gene (Maeso et al. 2016). A curious property of the genes is their high propensity for duplication and loss, and the subsequent variation in the gene repertoires possessed by different eutherian species (Maeso et al. 2016; Royall et al. 2018; Lewin et al. 2021) (fig. 1). While humans (*Homo sapiens*) have a complement similar to that of the eutherian ancestor, having lost only *PARGFX* and undergone no duplications, the ETCHbox cluster of mice (*Mus musculus*) is very different, with *ARGFX*, *DPRX*, and *PARGFX* completely lost, only remnants of *LEUTX* detectable and *TPRX1* (called *Crxos*) and *TPRX2* (known as *Obox*) both duplicated (Royall et al. 2018). Indeed, *M. musculus* possesses a huge array of 66 *Obox* loci (Royall et al. 2018). The ETCHbox repertoire of European cattle (*Bos taurus*) is more similar to that of human, except that *DPRX* is a pseudogene and there is an extra *TPRX* duplicate (Maeso et al. 2016; Lewin et al. 2021).

In addition to extensive gain and loss, ETCHbox genes exhibit a much faster rate of sequence change across the eutherians than do other homeobox genes (Maeso et al. 2016; Lewin et al. 2021). They are an archetypal example of asymmetric evolution, as their “parent” *CRX* has been conserved while ETCHbox sequences have changed drastically (Maeso et al. 2016; Katayama et al. 2018; Royall et al. 2018; Lewin et al. 2021). These features are unusual for homeobox genes, which are generally highly conserved because they perform key roles in embryonic development and tissue patterning (Bürglin and Affolter 2016). ETCHbox genes, therefore, pose a conundrum: they show extensive evolutionary change in gene number and amino acid sequence, yet they seem to have conserved temporal expression patterns and are hypothesized to possess key roles in the developing embryo. To resolve this paradox we need to know if similar expression is associated with similar and overlapping gene properties, and/or whether conserved expression between species is masking changes in gene functions driven by extensive amino acid sequence change.

To address these questions, we focus on *B. taurus* (European cattle) because its repertoire of ETCHbox genes is similar to that of the ancestral eutherian and to that of humans. This allows comparisons to be made between orthologues of *Bos* and human. Furthermore, since humans are within the clade Euarchontoglires and cattle are within Laurasiatheria, this comparison may permit insight into the ancient roles of ETCHbox genes in Eutheria.

There is also intrinsic interest in studying genes that may play regulatory roles in cattle embryonic development. Cattle are enormously important agriculturally: there are an estimated 1–1.5 billion head of cattle worldwide, almost 72 million tonnes of beef and buffalo meat, and 840 million tonnes of milk is produced annually, and demand may increase further if the human population rises as expected (Delgado 2003; FAO 2009, 2019; Ritchie and Roser 2017). It is, therefore, worrying that selective breeding in cattle has sometimes been associated with decreased fertility (Lucy 2001; Sheldon and Dobson 2003; Oltenacu and Algers 2005; Berry et al. 2014), and such infertility is a major cause of cattle culling worldwide (Ahlman et al. 2011; Ansari-Lari et al. 2012; Chiumia et al. 2013; Rilanto et al. 2020). Reduced fertility is caused in large part by a high failure rate of preimplantation embryos, the point at which ETCHbox genes are expressed, with up to 56% of cattle embryos lost before the blastocyst stage (Diskin and Morris 2008). The cost of early embryonic mortality in cattle was estimated at an annual $1.28 trillion worldwide (Perkel et al. 2015) and hence there is a clear imperative to better understand preimplantation development in cattle and identify genes and cellular processes that may be compromised in certain cattle breeds.

In this study, we aimed to examine the function of proteins encoded by ETCHbox genes of cattle, and compare these to their orthologues in humans. The timing and location of ETCHbox gene expression makes them particularly challenging to study in their native setting, and previous work has, therefore, utilized ectopic expression to gain functional insights (Madissoon et al. 2016; Maeso et al. 2016; Royall et al. 2018). This approach, in which the homeobox genes are expressed in cultured cells, asks whether the encoded proteins can elicit changes to cells in which they are not normally expressed. Since homeobox genes encode transcription factors, this can be assayed by analyzing the transcriptome after ectopic expression, a method which has proved highly informative. In this work, we utilize transcriptomic analysis after ectopic expression to understand the function of ETCHbox genes in *B. taurus*, characterize a mutant allele of *B. taurus ARGFX*, and test whether functional roles have changed during mammalian evolution.

## Results

### Transfection of Homeobox Genes into Bovine Fetal Fibroblasts

**Fig. 2.**
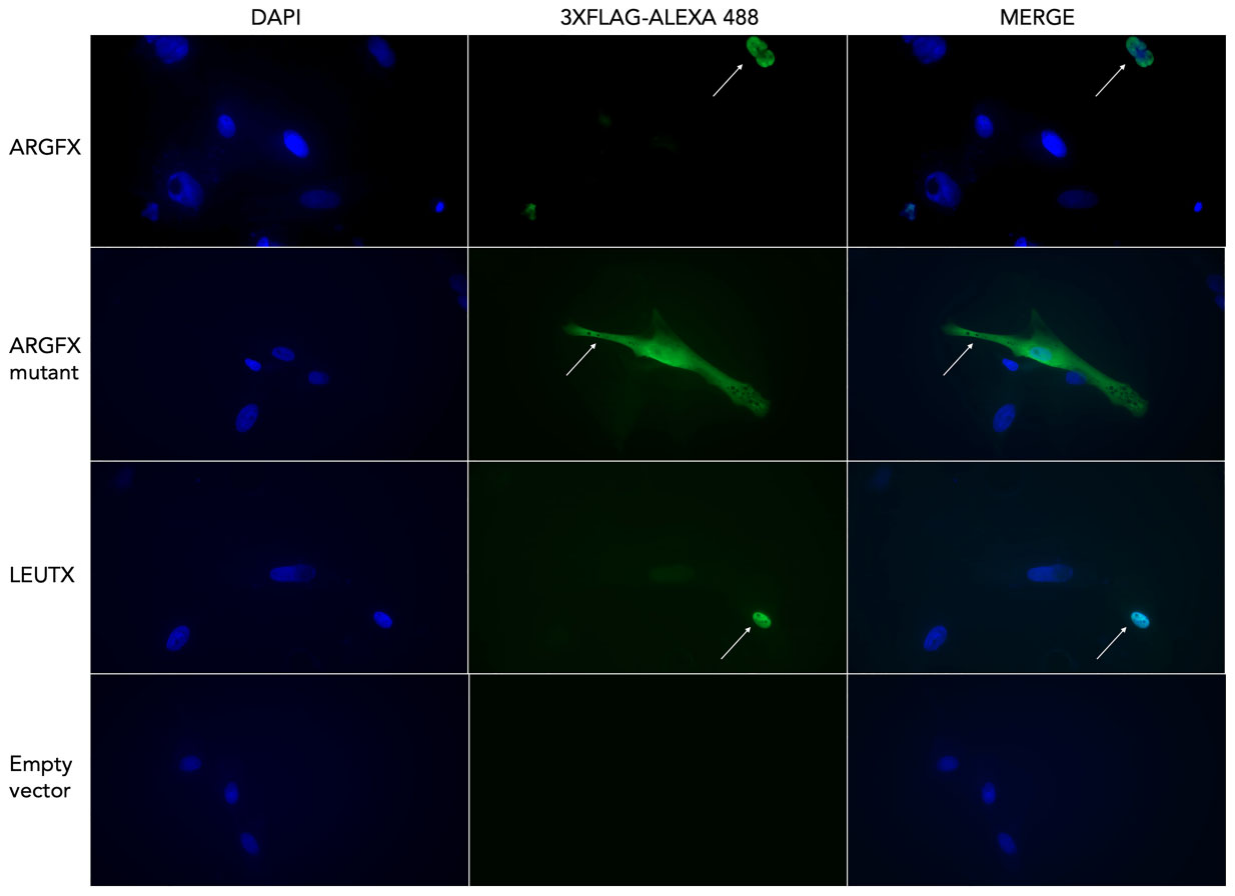
Immunocytochemistry analysis of BFF cells transfected with bovine ETCHbox genes. White arrows indicate cells with high expression of ETCHbox proteins. DNA is stained with DAPI (blue) to show cell nuclei. ETCHbox proteins are detected using Alexa Fluor 488-labelled antibodies to anti-FLAG antibodies (green). WT ARGFX and LEUTX proteins are localized to the nucleus; the mutant ARGFX is detected in the nucleus and cytoplasm. No green fluorescence is detected in samples transfected with the control plasmid.

In previous work, human ETCHbox gene function was studied by analyzing transcriptomic changes induced by ectopic expression in cultured fibroblasts, in which the genes are not natively expressed (Maeso et al. 2016). To facilitate comparison between orthologous bovine and human ETCHbox genes, here we conducted similar experiments using bovine genes in bovine fetal fibroblast (BFF) cells. First, we established a primary culture of fibroblasts from a biopsy of *B. taurus* fetal skin (supplementary fig. S1, Supplementary Material online). Second, we constructed expression plasmids carrying bovine homeobox genes *ARGFX* or *LEUTX* driven by a constitutive promoter, each connected by a peptide linker to a C-terminal 3xFLAG tag to facilitate downstream analysis. We also constructed an expression plasmid carrying a mutant allele of *ARGFX* identified in the ARS-UCD1.2 reference genome (Lewin et al. 2021). This allele has a 13 base pair (bp) deletion before the homeodomain resulting in a frameshift and truncated predicted protein. Immunocytochemistry showed that bovine ARGFX and LEUTX proteins both localize to the nucleus when transfected into cells, as expected for transcription factors (fig. 2). The mutant ARGFX protein was detected in the nucleus but was also abundant in the cytoplasm, suggesting the presence of a weaker nuclear localization signal. Control transfections were negative.

### Transcriptomic Changes Induced by Bovine Homeobox Genes

**Fig. 3.**
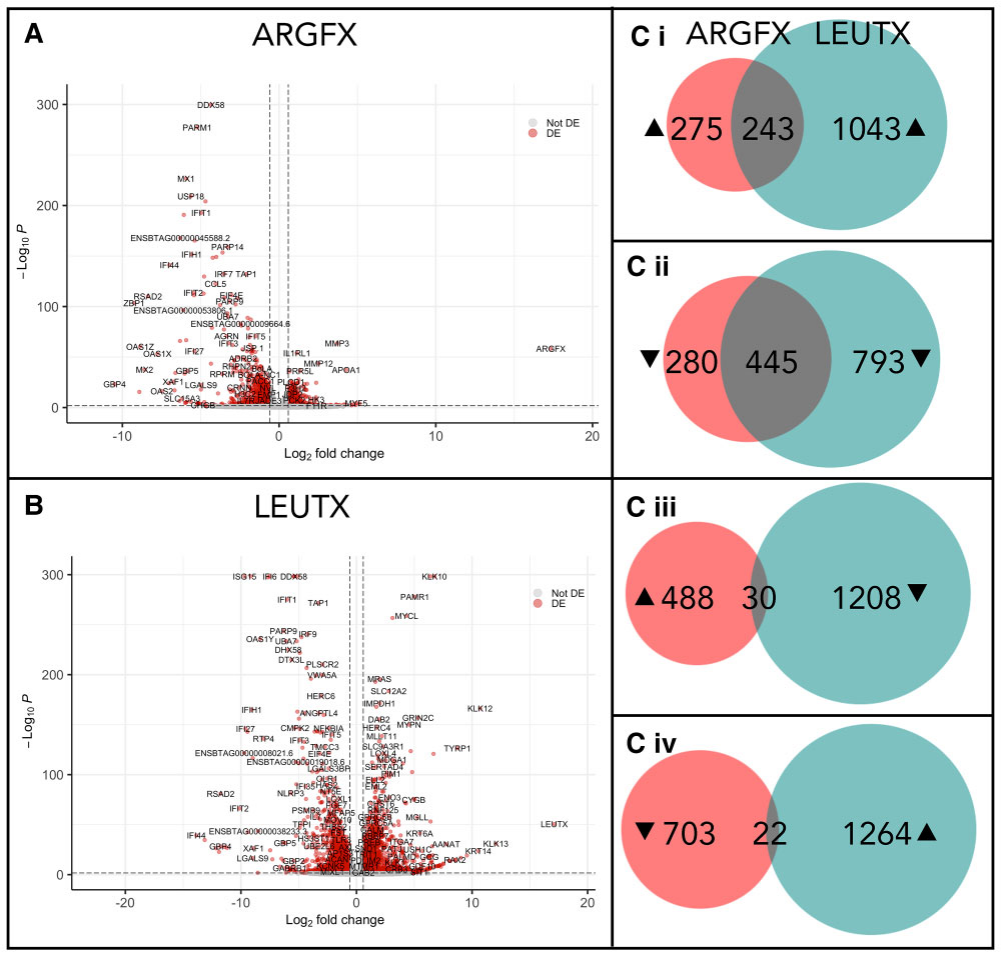
Transcriptional response to ETCHbox gene ectopic expression. Volcano plots showing genes DE in response to wild type (WT) (*A*) *ARGFX* and (*B*) *LEUTX* ectopic expression. Each point represents a gene. Points in red are considered DE (adjusted *P* < 0.05, fold change [FC] > 1.5). (*C*) Overlaps between sets of genes DE in response to *ARGFX* and *LEUTX* ectopic expression: (i) *ARGFX* (red) up, *LEUTX* (blue) up; (ii) *ARGFX* down, *LEUTX* down; (iii) *ARGFX* up, *LEUTX* down; and (iv) *ARGFX* down, *LEUTX* up.

We used transcriptomic analysis to determine whether ectopic expression of bovine *ARGFX* and *LEUTX* elicited gene expression changes in cells (supplementary table S1, Supplementary Material online). After transfection, RNA-sequencing (RNA-seq) followed by differentially expressed (DE) gene analysis identified which loci in the genome increased or decreased in expression level (fig. 3*A* and *B*; supplementary table S2*a*–*f*, Supplementary Material online). We found 518 and 725 genes up- and downregulated, respectively, following *ARGFX* ectopic expression, which likely includes direct and indirect targets. *LEUTX* expression caused changes to a larger number of genes: 1,286 increased in expression level and 1,238 decreased. *ARGFX* expression downregulates more genes than it upregulates, and the genes that decreased in expression after *ARGFX* transfection had a greater average fold change and lower *P*-value than those that increased in expression (fig. 3*A*); this suggests that ARGFX primarily has a repressive role in this cellular context. We performed gene ontology (GO) analysis on the DE gene sets (supplementary table S3*a*–*d*, Supplementary Material online).

We find that 47% of genes upregulated following *ARGFX* ectopic expression are also upregulated after *LEUTX* expression; 61% of genes downregulated by *ARGFX* expression are also downregulated by *LEUTX*. These overlaps between genes upregulated after *ARGFX* and *LEUTX* transfection (Fisher’s exact test *P* = 1.492 × 10^−90^) or downregulated in response to both genes (*P* = 5.176 × 10^−251^) are much greater than overlaps for genes affected antagonistically by *ARGFX* and *LEUTX*, for which there are no significant overlaps (6% genes upregulated in response to *ARGFX* are downregulated in response to *LEUTX*, *P* = 1.000; 3% genes downregulated in response to *ARGFX* are upregulated in response to *LEUTX*; *P* = 1.000) (fig. 3*C*). These analyses suggest a large degree of overlap in the cellular roles of bovine ARGFX and bovine LEUTX: the proteins perform similar functions rather than acting antagonistically. This is in contrast to data from humans, in which ARGFX and LEUTX act in opposite directions on the same group of genes (Maeso et al. 2016).

### Properties of Bovine, Human, and Mouse Homeobox Genes

The comparisons above suggest that bovine and human ETCHbox genes may not be performing identical roles. To investigate further, we compiled lists of genes DE in response to ETCHbox expression in three systems: bovine genes in bovine fetal dermal fibroblasts (this work), human genes in human dermal fibroblasts (Maeso et al. 2016), and mouse genes in mouse embryonic fibroblasts (Royall et al. 2018). We restricted lists of downstream genes to 1:1 orthologues; our dataset includes 21,666 bovine genes, of which 71.7% (15,534) had identifiable 1:1 orthologues in human and 71.6% (15,503) in mouse. Fisher’s exact test was then used to examine overlaps: this analysis asks whether overlap in the function of two genes is significantly greater than expected by chance. Indeed, many of the tests give apparently significant overlaps, hence we compared negative logs of adjusted *P*-values, which we call “overlap scores,” to extract biologically meaningful information: the higher the overlap score, the more the overlap exceeds the expected similarity. To provide context, previous work suggests that in humans ARGFX acts antagonistically to LEUTX and TPRX1, which have overlapping roles (Maeso et al. 2016).

**Fig. 4.**
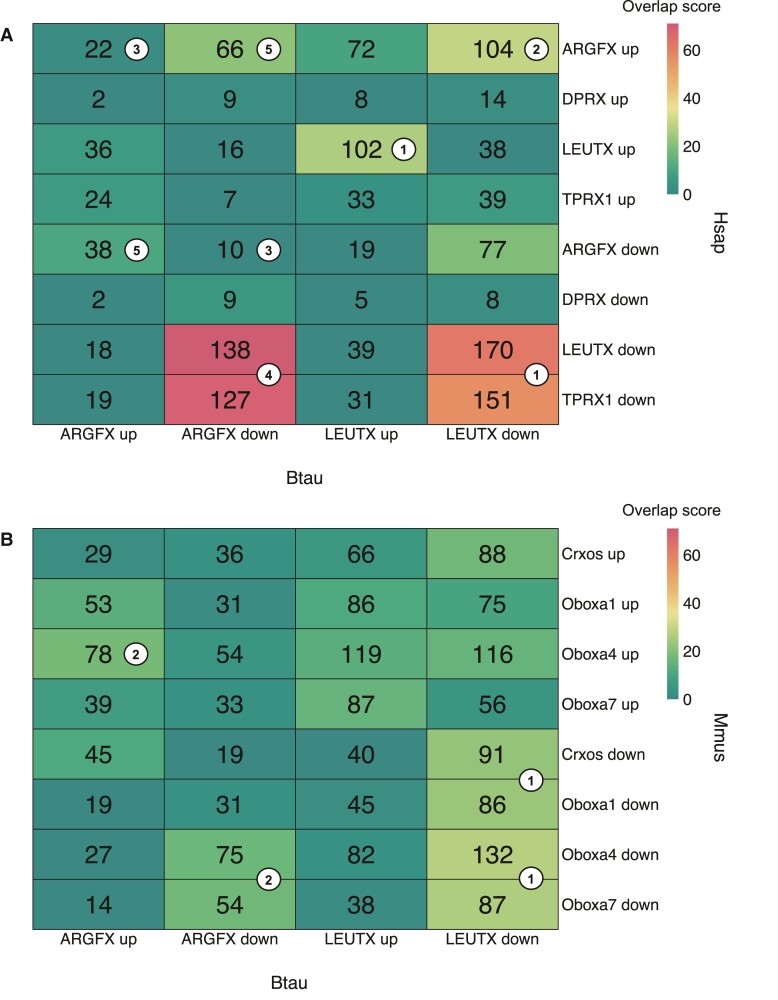
Overlap between genes DE in response to ectopic expression of (*A*) bovine and human and (*B*) bovine and mouse ETCHbox genes. For each comparison, numbers in the heatmap show the number of shared DE genes. Heatmap color scale reflects overlap scores. The greater the overlap score, the more the overlap of regulated genes exceeds that which is expected by chance. Overlap scores consist of −log_10_ multiple comparison-adjusted *P*-values from Fisher’s exact test for overlaps between gene sets. White dots with numbers mark points referred to in the main text. Btau, *Bos taurus*; Hsap, *Homo sapiens*; Mmus, *Mus musculus*.

Considering genes DE in response to bovine *LEUTX* expression, the highest overlap scores are for overlaps between: bovine *LEUTX* down (‘down’ referring to downstream genes that decrease in expression) genes and human *LEUTX* down genes (overlap score = 62.032), bovine *LEUTX* down genes and human *TPRX1* down genes (overlap score = 56.611), and then bovine *LEUTX* up (‘up’ referring to downstream genes that increase in expression) genes and human *LEUTX* up genes (overlap score = 26.833) (fig. 4*A*, points marked “1”). For example, 170 of the 1,019 bovine *LEUTX*-downregulated genes remaining after restriction to 1:1 orthologues are also downregulated by human *LEUTX*. Our test seeks to ask if this number is greater than expected by chance, and we conclude that it is (Fisher’s exact test *P* value = 9.28 × 10^−63^); from this, we deduce some conservation of function. Overall, these data suggest that *at least some aspects of the function of LEUTX are similar between humans and cattle*. Bovine *LEUTX*-downregulated genes also have a large overlap (overlap score = 29.979) with human *ARGFX* upregulated genes (fig. 4*A*, point marked “2”).

Considering genes downstream of bovine *ARGFX*, we find minimal overlap with gene sets affected by human *ARGFX*, suggesting *bovine and human ARGFX have different properties* (fig. 4*A*, points marked “3”). Larger overlaps, indeed the most significant overlaps in the whole study, are found when bovine *ARGFX* is compared with human *LEUTX* or *TPRX1*: bovine *ARGFX* down genes and human *LEUTX* down genes (overlap score = 70.383); bovine *ARGFX* down genes and human *TPRX1* down genes (overlap score = 68.166) (fig. 4*A*, points marked “4”). This suggests *that ARGFX in cattle has similar properties to LEUTX and TPRX1 in humans*. There are also large overlaps between bovine and human *ARGFX* in opposite directions: bovine *ARGFX* down genes and human *ARGFX* up genes (overlap score = 21.460); bovine *ARGFX* up genes and human *ARGFX* down genes (overlap score = 10.320) (fig. 4*A*, points marked “5”). While this final comparison has a smaller overlap score, it is still the highest overlap for bovine *ARGFX* upregulated genes with any human gene. This suggests that *bovine ARGFX acts in the opposite direction to human ARGFX*.

Comparing bovine and murine genes, the highest similarity of lists of DE genes is between bovine *LEUTX* down and mouse *Crxos*, *OboxA1*, *Oboxa4*, and *Oboxa7* down genes, but these similarities are smaller than bovine/human overlaps (overlap scores = 22.113, 23.008, 27.870, and 26.094, respectively) (fig. 4*B*, points marked “1”). This suggests that bovine ETCHbox genes are more similar in function to human ETCHbox genes than to those of mice. The highest overlap score for bovine *ARGFX* up genes is murine *Oboxa4* up genes (overlap score = 18.860) and for bovine *ARGFX* down genes is murine *Oboxa4* (overlap score = 16.250) and *Oboxa7* (overlap score = 17.984) down genes (fig. 4*B*, points marked “2”). These data suggest that both bovine ARGFX and LEUTX act in broadly the same direction as the tested mouse ETCHbox genes.

### Relevance of Target Genes to Bovine Development

The bovine *ARGFX* and *LEUTX* genes are expressed in a tight temporal window during preimplantation embryonic development (Maeso et al. 2016). To investigate if the gene expression changes induced by ectopic expression in fibroblasts have functional relevance, we employed temporal expression clustering. This approach tests whether the set of downstream target genes in fibroblast cells are enriched for genes normally expressed in specific temporal patterns in the embryo. Using published transcriptomic data, we determined temporal expression profiles for all bovine genes expressed from oocyte to blastocyst and clustered these into 100 groups sharing similar temporal expression profiles. We then tested whether the gene sets DE in response to *ARGFX* or *LEUTX* expression were enriched for genes in any expression profiles (supplementary table S4, Supplementary Material online).

**Fig. 5.**
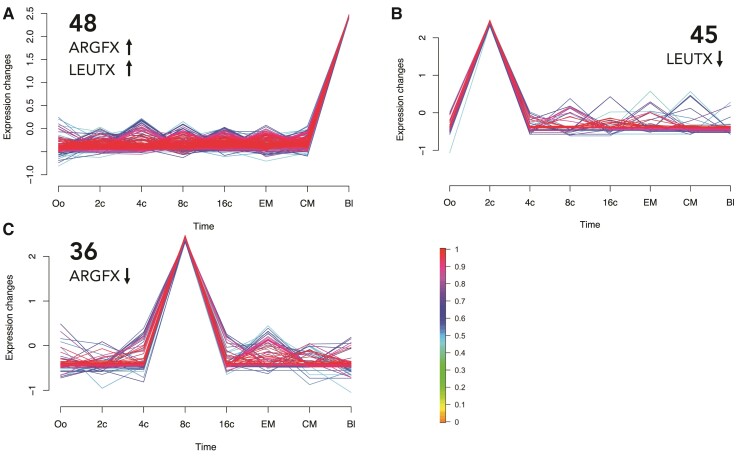
Temporal expression profiles for bovine genes enriched in the downstream target gene sets of ARGFX and LEUTX. Plots show normalized, standardized expression level as a function of developmental stage. Each line is the expression profile of one gene. Colors of lines show membership score from 0 to 1; membership score is a measure of how well the gene fits the profile. Genes with a score <0.5 were excluded from profiles. (*A*) Profile 48, *ARGFX* up and *LEUTX* up. (*B*) Profile 45, *LEUTX* down. (*C*) Profile 36, *ARGFX* down. Oo, oocyte; 2c, 2-cell; 4c, 4-cell; 8c, 8-cell; 16c, 16-cell; EM, early morula; CM, compact morula; Bl, early blastocyst.

Genes that increased in expression after ectopic expression of both *ARGFX* and *LEUTX* are enriched for profile 48 (Fisher’s exact test adjusted *P* = 0.005 and *P* = 0.041, respectively), which comprises genes that show minimal expression before the blastocyst but are then strongly expressed at the blastocyst stage (fig. 5*A*; supplementary fig. S2, Supplementary Material online). This supports the above conclusion that ARGFX and LEUTX functions are overlapping. The gene set downregulated by ectopic expression of *LEUTX* is enriched for genes belonging to expression profile 45 (Fisher’s exact test adjusted *P* = 0.005). This profile comprises genes expressed specifically at the two-cell stage (fig. 5*B*). Finally, profile 36 is enriched in the gene set downregulated following *ARGFX* ectopic expression (Fisher’s exact test adjusted *P* = 0.011); these genes are activated specifically at the 8-cell stage (fig. 5*C*).

We note that profile 48, upregulated by *ARGFX* and *LEUTX*, includes several genes with known roles in cell junctions and cell adhesion (*DSC2*, *EPDR1, FLRT2*), the cytoskeleton (*ACTG2, EPS8L2, HCK, MIB2, MYADM, PALLD, PLD2, SLC9A3R1, SMTN*), or the extracellular matrix (*COL15A1*, *COL5A2*, *COL5A3*, *COL6A3*, *CTHRC1*, *PXDN*) (supplementary fig. S2, Supplementary Material online), functions which are central to embryo compaction and the physical formation of the blastocyst. This suggests that bovine *ARGFX* and *LEUTX* regulate aspects of the structural formation of the blastocyst. Moreover, GO cellular component analysis of the shared gene set upregulated by *LEUTX* in both cattle and in humans (supplementary table S5, Supplementary Material online) shows extracellular matrix (GO:0031012) as an enriched function (*P* = 7.17 × 10^−4^), suggesting that this functional role may be conserved between the two species.

### ETCHbox Genes and Cell Potency

Induced pluripotent stem cells can be generated from mouse and human somatic cells by the addition of just four transcription factors: MYC, KLF4, POU5F1, and SOX2 (Takahashi and Yamanaka 2006; Takahashi et al. 2007). In this work, we find that bovine *ARGFX* and *LEUTX* both downregulate *MYC*, and *ARGFX* also downregulates *KLF4*. Inspecting DE gene lists from published works, we find that human *DPRX*, *LEUTX*, and *TPRX1* all downregulate *KLF4* and that four tested mouse ETCHbox genes (*Crxos*, *Oboxa1*, *Oboxa4*, *Oboxa7*) also downregulated *Sox2* when expressed in fibroblasts (Maeso et al. 2016; Royall et al. 2018). Furthermore, bovine *ARGFX* and *LEUTX* both downregulate the pluripotency-related gene *GBP4* and, indeed, the total bovine *LEUTX*-downregulated gene set is enriched for genes defining an “undifferentiation network signature,” which is composed of genes that are considered markers of an undifferentiated cell state (Galan et al. 2013) (Fisher’s exact test, *P* = 0.002; supplementary table S6, Supplementary Material online). Overall, both bovine *ARGFX* and *LEUTX* downregulate genes linked with an undifferentiated cell state.

### Frequency and Properties of a Mutant Bovine *ARGFX* Allele

**Fig. 6.**
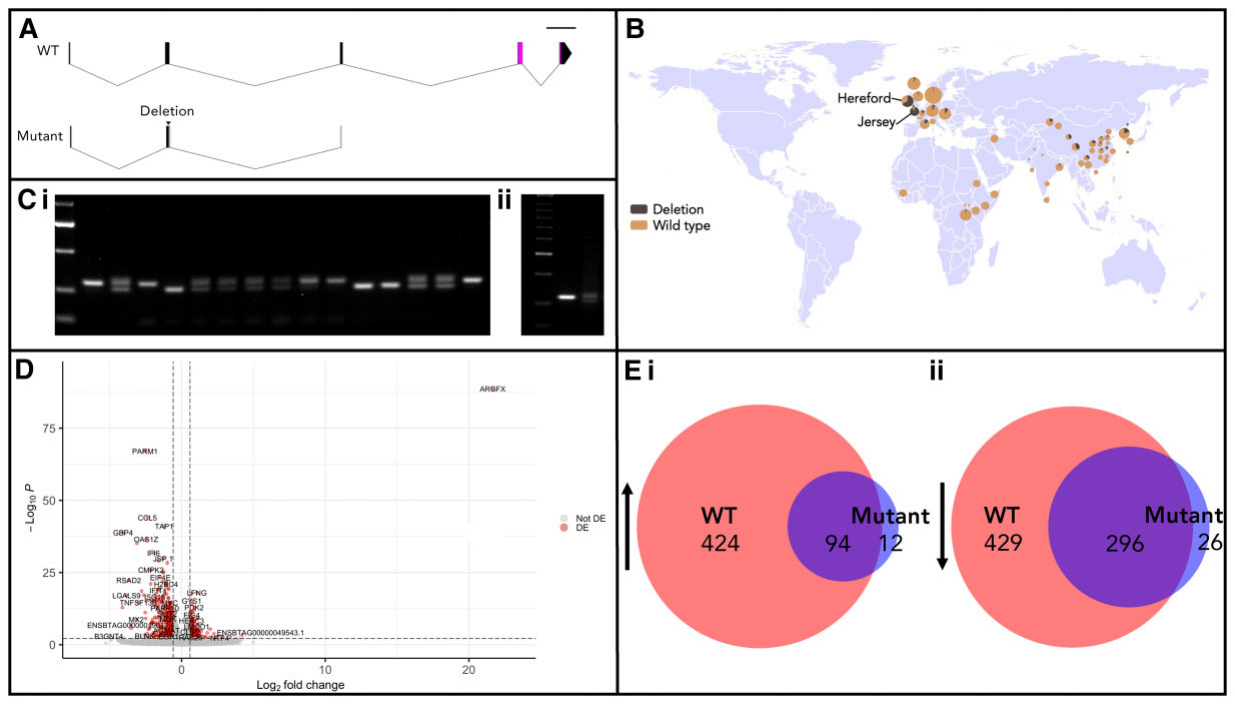
Characterization of a mutant bovine *ARGFX* allele. (*A*) Structure of WT and mutant genes and deduced proteins. The WT gene comprises five coding exons, with the homeobox (pink) split across exons 4 and 5. The mutant allele has a 13 bp deletion in exon 2 causing a frameshift (gray) and an early stop codon truncating the protein in exon 3 before the homeodomain. Scale bar 1 kb. (*B*) Map showing the distribution of the mutant allele. Dark brown/black = deletion, pale brown = WT. Pie charts show allele frequency for each breed; pie size proportional to sample size. Pie charts are located at the approximate location of the breed’s origin. Map generated using Bovine Genome Variation Database (Chen et al. 2020). (*Ci*) Agarose gel shows that individuals can be homozygous for the mutant *ARGFX* allele. PCR primers were used that anneal either side of the deletion site, producing a shorter amplicon in the deletion allele. Lane 1 = Bioline HyperLadder 50 bp. Lanes 2 and 16 = homozygous WT control. Lanes 3 and 15 = heterozygous control. Lanes 4 and 11 are homozygous WT Jersey individuals, lanes 5, 12, and 13 are homozygous mutant Jersey individuals and lanes 6, 7, 8, 9, and 14 are heterozygous Jersey individuals. Lane 10 is inconclusive, with a strong WT band and very weak mutant band. (*Cii*) Agarose gel showing that BFF cells have a WT *ARGFX* gene. Lane 1 = Bioline HyperLadder 50 bp. Lane 2 = BFF band. Lane 3 = heterozygote bands. (*D*) Volcano plot showing genes DE in response to *ARGFX* mutant ectopic expression. Each point represents a gene. Points in red are considered DE (adjusted *P* < 0.05, fold change [FC] > 1.5). (*E*) Overlaps between sets of genes DE in response to WT or mutant *ARGFX* ectopic expression: (i) upregulated by both and (ii) downregulated by both.

We previously identified a 13 bp deletion in the coding region of the *ARGFX* gene of the *B. taurus* reference genome ARS-UCD1.2 and showed that the indel is polymorphic, as several RNA-seq datasets exhibit the wild type (WT) allele (Lewin et al. 2021). Here, we investigate the frequency of the deletion allele and its effect on protein function. The mutation, a 13 bp deletion in exon 2, causes a frameshift that eliminates the homeodomain and truncates the protein (fig. 6*A*). We used the Bovine Genome Variation Database (Chen et al. 2020), which includes data from 432 *B. taurus* and *B. indicus* samples from 54 breeds, to assess the distribution of the two alleles. We find 25 of 54 (46%) breeds have at least one sampled individual with the mutant allele, and the limited sampling of some breeds suggests that the incidence may be higher. The reported mutant allele frequency is 0.122 across all samples and is highest in Jersey (0.917, 12 samples) and Hereford cattle (0.667, 21 samples) (fig. 6*B*; supplementary table S7, Supplementary Material online). Consistent with this, the reference genome ARS-UCD1.2 is from a Hereford individual. The deletion is present at least in low frequency in breeds originating from Africa, Asia, and Europe, and is found in both *B. taurus* and *B. indicus*. No other indels in the coding sequence of *ARGFX* are identified in the 432 samples.

To test whether individuals homozygous for the mutant *ARGFX* allele can be viable, we used polymerase chain reaction (PCR) to screen samples from the Jersey breed. From 11 individuals, we found three that are homozygous for the mutant allele, demonstrating that the homozygous mutant is not lethal (fig. 6*C*). Five individuals were heterozygous, two were homozygous WT, and one sample was inconclusive. We sequenced the *ARGFX* gene of all three homozygous mutant samples to confirm that the deletion found by PCR in Jersey cattle is the same 13 bp deletion identified in the reference genome. We also used PCR to genotype the BFFs used for ectopic expression; these were homozygous for the WT *ARGFX* allele (fig. 6*C*).

Analysis of RNA-seq data (Graf et al. 2014) from embryo pools containing both alleles shows that *ARGFX* continues to be expressed from a mutant locus. To understand the impact on protein function, we generated an expression construct containing the mutant *ARGFX* allele, transfected this into bovine fibroblasts, conducted transcriptome sequencing, and compared the sets of genes that changed in expression in response to transfection of WT or mutant *ARGFX* (fig. 6*D*). We found that the mutant allele has a much smaller set of downstream effects than does WT *ARGFX*, but that the DE genes overlapped strongly with those regulated by WT *ARGFX*. Just 18% and 41% of genes up- and downregulated by WT *ARGFX*, respectively, were also regulated by the mutant *ARGFX* but 89% of the genes that increased in expression and 92% of those that decreased in expression after transfection of mutant *ARGFX* had the same response to transfection of WT *ARGFX* (fig. 6*E*). This suggests that the truncated mutant protein is able to perform a subset of the functions of WT ARGFX, but not additional functions. This retention of a limited function despite protein truncation may be facilitated by cofactors that bind to the intact part of the protein.

## Discussion

The ETCHbox genes are a group of eutherian-specific homeobox genes that exhibit several puzzling characteristics. They are homeobox genes with a highly specific, conserved temporal expression pattern in the preimplantation embryo, and which are inferred to play roles at key developmental milestones such as embryonic genome activation (Jouhilahti et al. 2016; Madissoon et al. 2016; Maeso et al. 2016). Given this, one might expect them to be highly conserved but, in reality, the genes display rapid sequence evolution, and are also duplicated and lost at an unusually high rate (Maeso et al. 2016; Royall et al. 2018; Lewin et al. 2021).

We utilized ectopic expression to examine the roles of ETCHbox genes in cattle, and find evidence for large functional overlap between bovine *ARGFX* and *LEUTX*. This is consistent with the hypothesis that there is partial redundancy between ETCHbox proteins, and that this is one of the causes of the genes’ unusual evolutionary patterns (Lewin et al. 2021). Such redundancy may also explain why a frameshift mutation of *ARGFX* is not lethal when homozygous and has risen to a population allele frequency of over 10%; however, we stress that we do not know if homozygous mutant embryos have the same rate of survival as those with a fully functional copy of *ARGFX*. Regardless, there would be reduced selection against a suboptimal allele if gene function in the preimplantation embryo is redundant with *LEUTX*. Indeed, of the 424 ARGFX up genes and 429 ARGFX down genes which the mutant ARGFX protein fails to properly regulate, 45% and 48%, respectively, are also regulated in the same direction by LEUTX. This provides further evidence that LEUTX is able to directly compensate for the presence of a truncated ARGFX protein. Such redundancy may underpin why ETCHbox genes are lost in evolution at an elevated rate and, in this example, we may be observing this process in action: redundancy between two ETCHbox genes allowing one to move toward pseudogenization.

To test whether ETCHbox gene sequence divergence is accompanied by functional change, we compared the transcriptional response to ectopic expression between humans and cattle. Human and bovine ETCHbox orthologues share 48–77% amino acid sequence identity in their homeodomains and 36–54% identity across the full protein. We were surprised to find that *B. taurus* and *H. sapiens ARGFX* genes show minimal functional overlap, and that *B. taurus ARGFX* likely performs a function similar to that of *H. sapiens LEUTX*, and opposite to that of *H. sapiens ARGFX.* The change in broad functional roles is not seen, however, for LEUTX proteins, which have highly similar effects in human and bovine cells. We also found that bovine ETCHbox genes regulate gene sets that are more similar to those regulated by human ETCHbox genes than murine ETCHbox genes; this is consistent with the greater similarity of bovine ETCHbox sequences to those of humans than those of mice (Royall et al. 2018). In interpreting these results, we note the caveat that the human and cattle experiments were similar but not identical: analysis of human ETCHbox genes used ectopic expression in adult cells and analyzed transcriptome change 48 h posttransfection (Maeso et al. 2016), the bovine experiment used fetal cells, with RNA extracted after 72 h. These differences might cause variation in the gene sets regulated. Overall, however, these data suggest that significant changes in functional roles are a feature of ETCHbox evolution.

We utilized temporal expression profiling of bovine embryonically expressed genes to gain insights into the function of ETCHbox genes in the preimplantation embryo. The logic to this analysis is that if, during normal embryonic development, bovine ETCHbox genes upregulate or downregulate suites of genes at a particular stage, this may be replicated when the genes are ectopically expressed in cell culture. We find one temporal profile enriched in both the *ARGFX* and *LEUTX* upregulated gene sets: this profile consists of genes that have minimal expression up until the blastocyst but are then strongly induced. We suggest, therefore, that bovine *ARGFX* and *LEUTX* are involved in blastocyst formation. Interestingly, many blastocyst genes that are upregulated by *ARGFX* and/or *LEUTX* possess known roles in cell junctions and cell adhesion, the cytoskeleton or the extracellular matrix (supplementary fig. S2, Supplementary Material online). These functions are critical to embryo compaction and the structural changes that occur during morula and blastocyst formation (Albertini et al. 1987; Vestweber et al. 1987; Lehtonen et al. 1988; Enders et al. 1990; Damsky et al. 1993; Koyama et al. 1994; Hardy et al. 1996; Fleming et al. 2000; Fleming 2001; Watson and Barcroft 2001; Eckert and Fleming 2008; Hasley et al. 2017; Aberkane et al. 2018; Lim and Plachta 2021); this suggests that ETCHbox genes are involved in coordinating the physical process of structural formation of the blastocyst. We note, however, that, despite GO analyses suggesting that this role may be conserved in humans, a similar response of “blastocyst genes” was not detected in experiments using human cells (Maeso et al. 2016). One possible explanation for the difference is that blastocyst gene expression could be a downstream effect, not observed in the human experiments when transfected cells were harvested sooner (Maeso et al. 2016). We do not find induction of the proinflammatory genes *S100A9* or *TNFA*, implicated in inducing an inflammation response in uterine cells at implantation (He et al. 2019).

The timing of ETCHbox genes’ expression in cattle and human, peaking just before blastocyst formation, and for *ARGFX* continuing into the blastocyst (Maeso et al. 2016), is consistent with the hypothesis that bovine and human ETCHbox genes are involved in coordinating the physical process of blastocyst formation. A function for ETCHbox genes in embryo compaction and the structural formation of the blastocyst is also consistent with their absence in the sister lineage to placentals, marsupials, which do not undergo compaction, possess a morula stage or form an inner cell mass as part of their blastocyst (Renfree and Lewis 1996; Renfree 2010; Renfree and Shaw 2014; Royall et al. 2019). The upregulation of the same temporal profile by both ARGFX and LEUTX supports the contention that bovine ARGFX and LEUTX have overlapping functions.

**Fig. 7.**
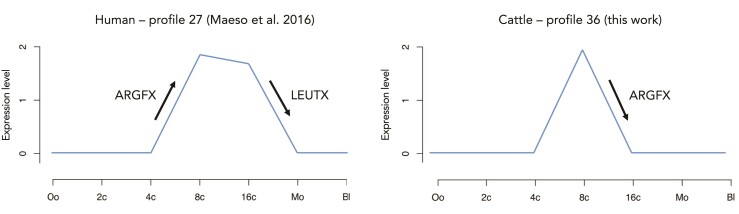
Temporal expression profiles for genes upregulated in response to *ARGFX* expression in human (Maeso et al. 2016) and downregulated in response to *ARGFX* expression in bovine (this work). Oo, oocyte; 2c, 2-cell; 4c, 4-cell; 8c, 8-cell; 16c, 16-cell; Mo, morula; Bl, early blastocyst.

The bovine *LEUTX*-downregulated gene set is enriched for genes expressed at the 2-cell stage, suggesting that *LEUTX* may downregulate the 2-cell program as part of the exit from the 2-cell stage. Intriguingly, *ARGFX* downregulates a temporal profile comprising genes expressed at the 8-cell stage. This profile is similar to that identified in previous work (Maeso et al. 2016) as downregulated by ectopic expression of human *LEUTX* and *TPRX1*, and upregulated by *ARGFX* (fig. 7); this supports our earlier conclusion that *B. taurus ARGFX* performs the opposite role to human *ARGFX* and is more similar functionally to human *LEUTX.* Overall, the temporal expression profiles enriched in the gene sets regulated by ectopic expression of ETCHbox genes are consistent with the timing of ETCHbox expression in cattle development, indicate that the ectopic expression experiments lead to the activation of realistic embryonic targets, and suggest that ETCHbox genes are involved in blastocyst formation.

We find that human and bovine ETCHbox genes downregulate the expression of Yamanaka factors, and that the total bovine *LEUTX*-downregulated gene set is enriched for genes of the “undifferentiation network signature” of the early preimplantation embryo (Galan et al. 2013). Cells of the preimplantation embryo undergo a reduction in their potency as the totipotent blastomeres of early development undergo lineage specification and differentiation to form the blastocyst (Lu and Zhang 2015; Hu 2019); the downregulation of genes associated with an undifferentiated state is, therefore, consistent with the timing of ETCHbox genes’ expression just prior to blastocyst formation.

Finally, we also examined which gene targets had the strongest response to ETCHbox expression. Eight of the ten downregulated genes with the highest fold change were common between *ARGFX* and *LEUTX*; seven of these eight genes (*GBP4*, *IFI44*, *MX2*, *OAS1X*, *OAS1Z*, *RSAD2* [*VIPERIN*], *ZBP1*) are known to be interferon-stimulated genes (ISGs), which are regulated via the JAK/STAT signaling pathway (Indraccolo et al. 2007; DeFilippis et al. 2010; Hu et al. 2011; Seo et al. 2011; Kane et al. 2013; Schneider et al. 2014; Vladimer et al. 2014; Hernandez et al. 2015; Harwardt et al. 2016; DeDiego et al. 2019; Kuepper et al. 2019; Tirumurugaan et al. 2020). Two more ISGs, *XAF1* and *IFIT2*, are in the top 10 downregulated genes for *ARGFX* and *LEUTX*, respectively (Sun et al. 2008; Zhou et al. 2013). Moreover, the overall gene sets downregulated in response to *ARGFX* and *LEUTX* expression are each enriched for six GO Biological Process terms related to the interferon response (table 1). Overall, these data suggest that the expression of ISGs is downregulated by bovine *ARGFX* and *LEUTX*.

**Table 1.** Interferon-related GO Terms Enriched in *ARGFX* and *LEUTX*-Downregulated Gene Sets.

Interferon-related GO term	Bovine *ARGFX* FDR-adjusted *P*-value	Bovine *LEUTX* FDR-adjusted *P*-value	Bovine *LEUTX* and Human *LEUTX* Overlap FDR-adjusted *P*-value
Cellular response to interferon-beta (GO:0035458)	NS	NS	2.89 × 10^−3^
Cellular response to interferon-gamma (GO:0071346)	NS	NS	1.24 × 10^−3^
Cellular response to type I interferon (GO:0071357)	1.32 × 10^−2^	1.66 × 10^−2^	5.63 × 10^−4^
Interferon-gamma-mediated signaling pathway (GO:0060333)	NS	NS	1.31 × 10^−5^
Positive regulation of interferon-alpha production (GO:0032727)	NS	NS	1.28 × 10^−2^
Positive regulation of interferon-beta production (GO:0032728)	NS	NS	3.99 × 10^−6^
Positive regulation of type I interferon production (GO:0032481)	NS	NS	1.48 × 10^−6^
Regulation of interferon-alpha production (GO:0032647)	NS	NS	1.30 × 10^−3^
Regulation of interferon-beta production (GO:0032648)	NS	NS	1.34 × 10^−7^
Regulation of type I interferon production (GO:0032479)	NS	NS	3.63 × 10^−8^
Response to interferon-alpha (GO:0035455)	1.74 × 10^−3^	2.71 × 10^−3^	8.50 × 10^−3^
Response to interferon-beta (GO:0035456)	2.54 × 10^−4^	3.91 × 10^−3^	7.75 × 10^−5^
Response to interferon-gamma (GO:0034341)	4.69 × 10^−3^	1.18 × 10^−3^	5.27 × 10^−5^
Response to type I interferon (GO:0034340)	8.14 × 10^−4^	3.50 × 10^−3^	6.51 × 10^−7^
Response to type III interferon (GO:0034342)	NS	NS	3.40 × 10^−2^
Type I interferon signaling pathway (GO:0060337)	4.57 × 10^−2^	3.82 × 10^−2^	6.83 × 10^−3^

NOTE.—GO biological process terms related to interferon signaling enriched in gene sets downregulated by *ARGFX* and *LEUTX*. *P*-values are from binomial tests with FDR corrections for multiple testing. FDR, false discovery rate; GO, gene ontology; NS, not significant.

Through what mechanism are ETCHbox genes regulating ISGs? We suggest that ETCHbox genes are capable of downregulating the JAK/STAT pathway: we find that bovine *LEUTX* and/or *ARGFX* stimulate downregulation of expression of *JAK2*, *STAT1*, *STAT2* and upregulation of the JAK/STAT inhibitors *PIAS3* and *PIAS4* (supplementary table S2, Supplementary Material online). Since cell surface receptors compete for JAKs, meaning that JAK concentration is often a limiting factor for JAK/STAT signaling (Haan et al. 2006; Schneider et al. 2014), this downregulation of *JAK2* may result in a direct downregulation of JAK/STAT signaling potential. We note that there is also downregulation of the JAK/STAT inhibitors SOCS1-5; while this may initially seem counterintuitive, these are also ISGs and are upregulated by JAK/STAT, so a decrease in their expression is consistent with reduced JAK/STAT signaling (Matsumoto et al. 1997; Krebs and Hilton 2001; Bousoik and Montazeri Aliabadi 2018). The JAK/STAT signaling pathway plays important roles in preimplantation development at the time of normal ETCHbox expression. For example, in mice the pluripotency factor POU5F1 acts via JAK/STAT3 signaling to induce pluripotency (Stirparo et al. 2021), and JAK/STAT3 signaling alone can induce naïve pluripotency during reprograming (Yang et al. 2010; Van Oosten et al. 2012). In cattle, the JAK/STAT pathway is vital for inner cell mass gene expression and development (Meng et al. 2015). Indeed, it has been found that JAK/STAT signaling is essential for pluripotency maintenance, self-renewal and the prevention of differentiation (Niwa et al. 1998; Nichols et al. 2001; Do et al. 2013; Stirparo et al. 2021); the downregulation of JAK/STAT signaling is, therefore, consistent with the above result that genes involved in the maintenance of an undifferentiated cell state are also downregulated by ETCHbox genes. Together with the temporal profile analyses, these data suggest that ETCHbox genes are involved in the promotion of blastocyst structural formation, and downregulate genes involved in the undifferentiation network in cells, possibly via the downregulation of JAK/STAT signaling. GO terms related to interferon signaling are strongly enriched in the set of genes commonly downregulated by human and bovine *LEUTX* (table 1 and supplementary table S8, Supplementary Material online); this suggests that this function is conserved between humans and cattle.

## Conclusions

Fast-evolving homeobox genes pose a conundrum in developmental biology since they contrast so markedly from the evolutionary conservation typical of homeobox genes. In this work, we utilized an ectopic expression approach to compare the functions of bovine ETCHbox genes to those of humans and mice and found evidence suggesting that, remarkably, the function of bovine *ARGFX* is highly different to that of its human orthologue and is more similar to that of human *LEUTX.* This suggests that the gene’s function has undergone a transition since the divergence of humans and cattle. In addition, we found that bovine *ARGFX* and *LEUTX* upregulate blastocyst-expressed genes, including genes involved in cell adhesion, the extracellular matrix and the cytoskeleton; we suggest that these genes have overlapping roles in orchestrating structural changes during blastocyst formation. Our results also suggest that ETCHbox genes downregulate JAK/STAT signaling, and that this may result in the downregulation of genes associated with an undifferentiated cell state. Finally, the existence of a frameshift allele that truncates the bovine ARGFX protein and eliminates many of its functions, but is nonlethal when homozygous, supports the hypothesis of redundancy between ETCHbox proteins.

## Materials and Methods

### Cell Isolation and Culture

Primary BFFs were isolated from the torso skin of a 6-month-old *B. taurus* fetus from a pregnant female sacrificed at an abattoir, using a method similar to that used to isolate human dermal fibroblasts (Vangipuram et al. 2013). Briefly, a 1 cm^2^ section of skin was excised, transported on ice and washed 3 times with sterile phosphate-buffered saline (PBS) (Gibco #10010015). Subcutaneous fat was scraped off and the tissue was minced with a scalpel. Five 2 mm^2^ pieces were placed in each well of a 0.001% poly-l-lysine-coated (Sigma-Aldrich, #P4832-50ML) 6-well plate, covered with a sterile glass coverslip, and 2 ml BFF media added. BFF media is Dulbecco’s modified Eagle medium (Gibco #41965039) with 10% heat-inactivated fetal bovine serum (Gibco #10500064), 1% penicillin-streptomycin (Gibco #15140122), and 1% amphotericin B (Gibco #15290026). Outgrowth was allowed for 18 days at 37 °C with 5% CO_2_, with the culture media changed every 3 days before cells were washed three times in 2 ml PBS and detached with 2 ml TrypLE Express enzyme (Gibco #12604013) at 37 °C with 5% CO_2_ for 20 min. TrypLE Express was neutralized with 5 ml BFF medium, the coverslip removed, cells and medium transferred to a 50 ml Falcon tube and centrifuged for 5 min at 1,200 rpm. Cells were washed twice with 10 ml BFF medium and centrifuged, then seeded into 0.001% poly-l-lysine-coated T75 flasks. To maintain growth, fibroblasts were incubated at 37 °C with 5% CO_2_ and passaged one flask to four every 3–4 days at 70–80% confluency. Genomic DNA was extracted using a DNeasy Blood and Tissue Kit (Qiagen #69504), and the *cytochrome c oxidase subunit I* gene amplified by PCR (primer pair 1; supplementary table S9, Supplementary Material online) and sequenced to verify species identity of the cultured cells (supplementary fig. S3, Supplementary Material online). Cells were tested and negative for mycoplasma contamination.

### Ectopic Expression

*Bos taurus ARGFX* and *LEUTX* intron/exon structures were determined previously (Lewin et al. 2021). Where multiple isoforms were present, the isoform with the highest FPKM (Fragments per kilobase of transcript per million mapped reads) value and possessing an exon structure matching that of the human gene was used. *ARGFX* and *LEUTX* complete coding sequences with a C-terminal GGGGSGGGGS linker and 3xFLAG were synthesized by ThermoFisher GeneArt and Twist Bioscience, respectively (supplementary fig. S4, Supplementary Material online). Sequences were cloned into plasmid pSF-CMV-Puro-COOH (OXGENE #OG3422) under the control of a CMV promoter using XhoI (Thermo Scientific #FD0695) and NotI (Thermo Scientific #FD0593) (primer pair 2). An *ARGFX* mutant containing the 13 bp deletion found in the ARS-UCD1.2 reference genome was produced using a Q5 Site-Directed Mutagenesis Kit (New England BioLabs #E0554S). The deletion was induced with primer pair 3, then primer pair 4 used to replace the premature stop codon caused by the frameshift with the GGGGSGGGGS linker and in-frame 3xFLAG. Constructs were validated by Sanger sequencing (primer pair 5).

Cells were transfected with either the WT bovine *ARGFX* gene, *LEUTX* gene, mutant *ARGFX* gene or empty pSF-CMV-Puro-COOH vector as a control. For transfection, 1 million cells (passage <6) were resuspended in 98 μl Opti-MEM (Gibco #11058021), combined with 2 μl of 5 μg/μl endotoxin-free plasmid, electroporated with a 2 mm gap using a NEPA21 Super Electroporator (NEPAGENE) and seeded in 2 ml BFF medium. The medium was changed after 24 h, and puromycin (Sigma Aldrich #P8833) added at 4 μg/ml to select for transfected cells. After 72 h, RNA was extracted using an RNeasy Plus Micro kit (Qiagen #74034). RNA purity was measured using a Nanodrop ND-1000 spectrophotometer and RNA integrity checked using an Agilent 2100 Bioanalyzer.

For one sample of each construct, immunocytochemistry was undertaken to check for protein expression using an antibody to 3xFLAG, as described in previous work (Maeso et al. 2016) with the following differences: primary antibody = monoclonal mouse anti-FLAG M2 antibody (Sigma Aldrich #F1804-50UG) 1:100, 4 h incubation time; secondary antibody = goat antimouse IgG (H + L) superclonal recombinant secondary antibody with Alexa Fluor 488 (Invitrogen #A28175) 1:1000, 1 h incubation time. Cells were mounted using SlowFade Gold Antifade Mountant with DAPI (Invitrogen #S36938) to stain DNA and visualized with a Zeiss Axioskop 2 Plus fluorescence microscope and Zeiss Axiocam 202 mono camera. Images were processed using ImageJ.

### Analysis of RNA-seq Data

RNA sequencing was performed on four biological replicates for each plasmid using the Illumina NovaSeq 6000 Sequencing System (Novogene), giving between 77.7 million and 120.9 million paired-end 150 bp reads per sample. Reads were filtered to remove those containing adapters, those with *N* > 10% and those of low quality (Qscore < 5). Quality control was performed using FastQC version 0.11.8 (Andrews 2010), MultiQC version 1.8 (Ewels et al. 2016), and Trimmomatic version 0.39 used to trim reads (Bolger et al. 2014). The pseudoaligner kallisto version 0.46.1 (Bray et al. 2016) was used to align reads to the *B. taurus* ARS-UCD1.2 transcriptome and quantify abundances; *B. taurus* ETCHbox sequences were added to the transcriptome before pseudoalignment. STAR version 2.7.9a (Dobin et al. 2013), StringTie version 2.1.7 (Pertea et al. 2015), and the Integrative Genomics Viewer (Robinson et al. 2011) were used to align, assemble, and visualize reads of *ARGFX* and *ARGFX* mutant samples to verify that the correct version was expressed. R version 4.1.0 was used for data analysis (R Core Team, 2020). Tximport version 1.20.0 was used to create count tables (Soneson et al. 2016), and DESeq2 version 1.32.0 used for DE analysis (Love et al. 2014). The false discovery rate (FDR = 0.05) method was used to correct for multiple testing. Genes with an adjusted *P*-value < 0.05, fold change > 1.5, and mean TPM (transcripts per million) > 2 were considered DE. One of the four *ARGFX* mutant samples was excluded as it was an outlier on PCA plots, clustering with *ARGFX* WT samples (supplementary fig. S5, Supplementary Material online). Volcano plots were created using R package EnhancedVolcano version 1.10.0 (Blighe et al. 2021) and Venn diagrams with BioVenn (Hulsen et al. 2008). GO analysis (Ashburner et al. 2000) was completed using PANTHER version 16.0 (Thomas et al. 2003; Mi et al. 2013, 2021) using binomial tests and an FDR correction for multiple testing (FDR = 0.05).

We tested whether there were significant overlaps between the genes DE in response to the expression of *B. taurus*, *M*. *musculus*, and *H. sapiens* ETCHbox genes. To achieve this, we combined the bovine data from this work with lists of genes reported to be DE in response to *H. sapiens ARGFX*, *DPRX*, *LEUTX*, and *TPRX1* ectopic expression and mouse *Crxos*, *Oboxa1*, *Oboxa4*, and *Oboxa7* ectopic expression (Maeso et al. 2016, Royall et al. 2018). Gene lists were first restricted to 1:1 orthologues, identified using OMA (Altenhoff et al. 2021; supplementary table S10*a* and *b*, Supplementary Material online). Fisher’s exact test was then used to test for overlaps in the gene sets up- or downregulated by bovine *ARGFX* or *LEUTX* and each of either the human or mouse ETCHbox genes. Many of these tests produce significant overlaps so, to identify the “most significant” overlaps and facilitate clearer comparisons, we calculated an overlap score consisting of the −log_10_ of the multiple comparison-corrected *P*-value. The greater the overlap score, the more the overlap in the genes’ activity exceeds that expected by chance. For these tests, the Benjamini-Yekutieli FDR method (Benjamini and Yekutieli 2001) (FDR = 0.05) was used to correct for multiple testing because it does not assume independence of tests.

To gain insights into the role of bovine ETCHbox genes in the preimplantation embryo, temporal expression profile clustering was employed. To obtain a dataset for clustering, raw reads from a published bovine embryonic RNA-seq dataset (Jiang et al. 2014), covering oocyte, 2-cell, 4-cell, 8-cell, 16-cell, early morula, compact morula and blastocyst stages, were downloaded from NCBI SRA (PRJNA254699) and mapped with STAR (Dobin et al. 2013), then transcripts were quantified with StringTie (Pertea et al. 2015) as above. Mfuzz version 2.52.0 (Kumar and Futschik 2007) was then used to cluster bovine embryonically expressed genes into temporal expression profiles. The fuzzy c-means algorithm of Mfuzz was selected as it is more robust to the noise present in RNA-seq data than is k-means clustering. Clusters were restricted to genes with a membership value >0.5, and Fisher’s exact test was used to test for enrichment of genes of each cluster in the sets of genes DE in response to ETCHbox expression. The Benjamini and Hochberg (1995) FDR method (FDR = 0.05) was used to correct for multiple testing.

Fisher’s exact test with a Benjamini and Hochberg (1995) adjustment for multiple testing was used to test for enrichment of ‘undifferentiation’ marker genes in the lists of genes DE in response to bovine ETCHbox ectopic expression. The undifferentiation network signature consists of 266 genes (Galan et al. 2013), of which 234 had identifiable 1:1 orthologues in bovine.

### Frequency of an *ARGFX* Mutant Allele

The Bovine Genome Variation Database (Chen et al. 2020) was used to assess the allele frequency and distribution of a mutant *ARGFX* allele identified in the *B. taurus* ARS-UCD1.2 reference genome. To test whether individuals can be homozygous for the mutant form of *ARGFX* or whether it is lethal, PCR (GoTaq G2 DNA polymerase, Promega #M7841) was used to characterize the *ARGFX* gene of 11 individuals of the Jersey breed, which possess a high frequency of the mutant allele. Nasal fluid was collected from *B. taurus* individuals using GenoTube Livestock swabs (ThermoFisher Scientific #9062010). To extract genomic DNA, a 5 mm^2^ fragment was cut from the swab with sterile scalpels and minced, and then treated as a tissue sample for extraction using the DNeasy Blood and Tissue Kit (QIAGEN #69504). A primer set (pair 6) was designed with primers either side of the deletion, giving a 13 bp shorter amplicon for a mutant allele versus WT. To distinguish bands with a 13 bp difference in length, a 3% TBE (Tris–Borate–EDTA) gel was used. To test which form of *ARGFX* is present in the BFF cells, genomic DNA was extracted with a DNA Isolation Kit (Roche #11814770001) using the standard protocol, and the above primer sets used.

## Supplementary Material

Supplementary data are available at *Molecular Biology and Evolution* online.

## Supplementary Material

msac098_Supplementary_Data

## Data Availability

Raw and processed sequencing datasets have been deposited to the NCBI Gene Expression Omnibus (GEO) (www.ncbi.nlm.nih.gov/geo) under accession GSE192356.
