# Supplementary material for: PRD-Class Homeobox Genes in Bovine Early Embryos: Function, Evolution, and Overlapping Roles
Source: Mol Biol Evol. 2022 May 5;39(5):msac098. doi: 10.1093/molbev/msac098 (PMC9117796; doi:10.1093/molbev/msac098)
Supplement: msac098_Supplementary_Data [file msac098_supplementary_data.zip › ESM1 - Supplementary Figures.pdf]

Molecular Biology and Evolution

PRD-class homeobox genes in bovine early embryos: function, evolution and overlapping roles

Thomas D. Lewin[1], Ali A. Fouladi-Nashta[2] and Peter W. H. Holland[1*]

Affiliations:

[1]Department of Zoology, University of Oxford, 11a Mansfield Road, Oxford, OX1 3SZ, UK

[2]Comparative Biomedical Sciences Department, Royal Veterinary College, Hawkshead Campus, North Mymms, Hatfield, Hertfordshire, AL9 7TA, UK

Corresponding author: Peter W. H. Holland (peter.holland@zoo.ox.ac.uk)

Supplementary Figures S1 – S5

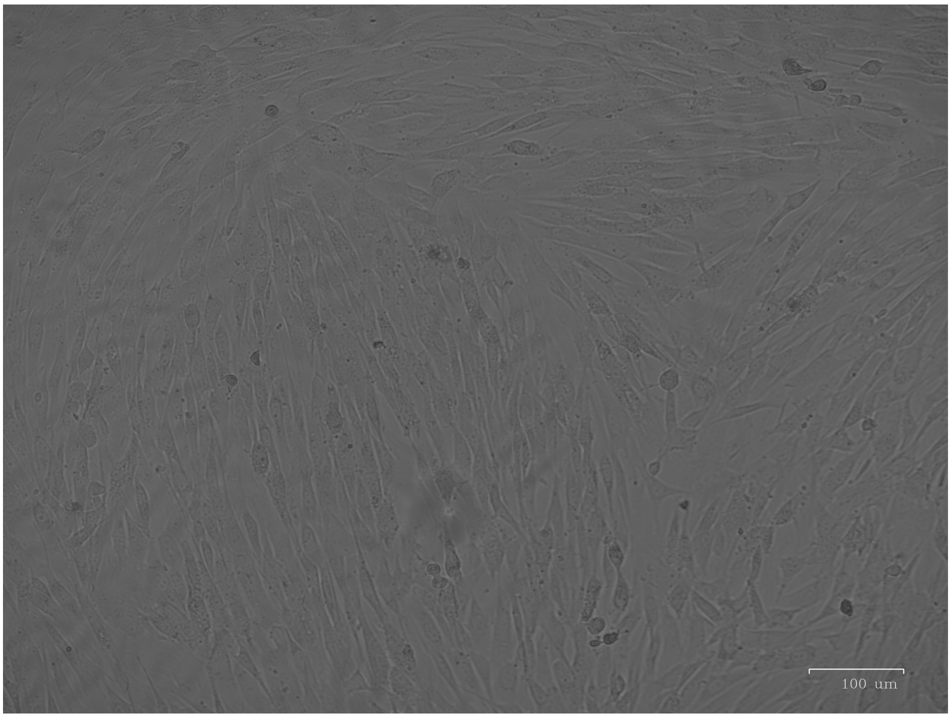

Supplementary Fig. S1 Image of primary bovine foetal fibroblast (BFF) cells at passage 3 post-isolation. Image captured with ZOE Fluorescent Cell Imager (Bio-Rad).

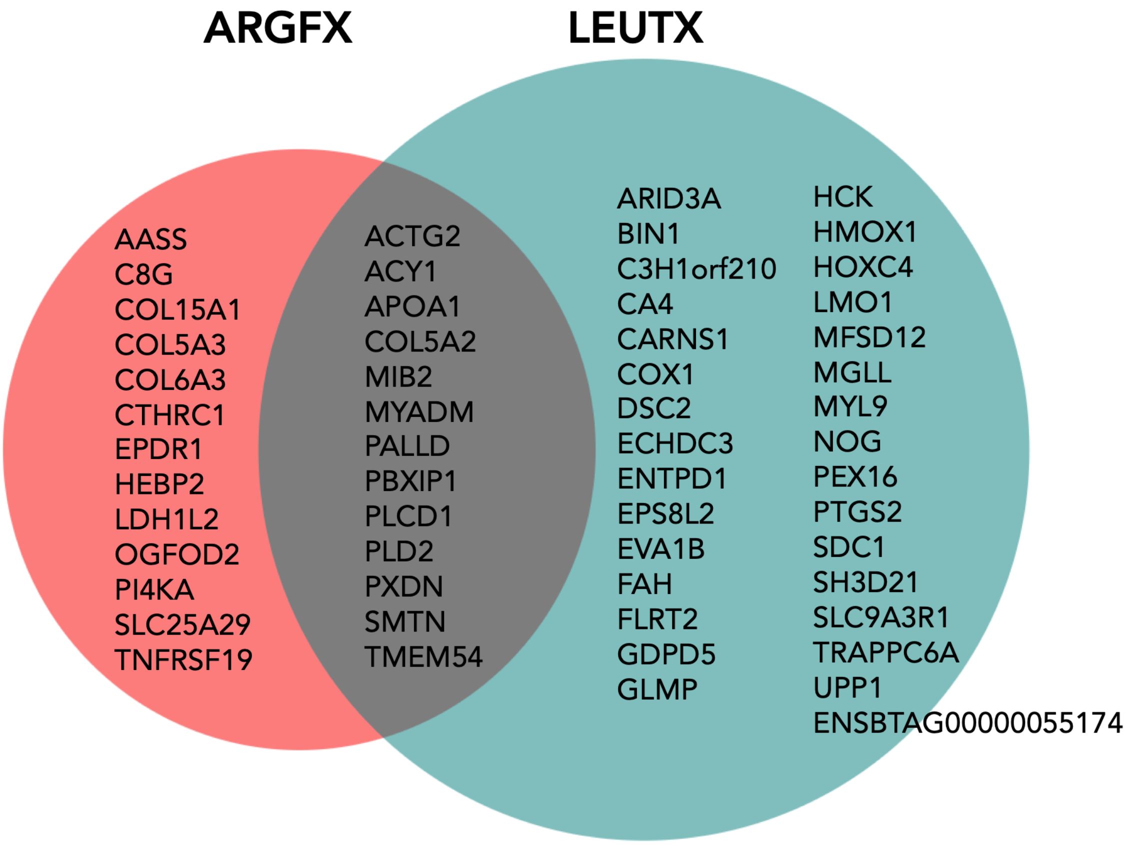

Supplementary Fig. S2 Blastocyst genes (profile 48) that are upregulated by *ARGFX*, *LEUTX* or both genes.

```
>BFF COI gene
TATGGCGATAGGCACCGGACTCTGCTCGGAGACGACCAAATCTACAACGTAGTNGTTNCGCA
CACGCATTTGTAATAATCTTCTTCATAGTAATACCAATCATAATTGGAGGATTCGGTAACTG
ACTTGTTCCCCTAATAATTGGTGCTCCCGATATAGCATTTCCCCGAATAAATAATATAAGCT
TCTGACTCCTCCCTCCCTCATTCCTACTACTCCTCGCATCCTCTATAGTTGAAGCTGGGGCA
GGAACAGGCTGAACCGTGTACCCTCCCTTAGCAGGCAACCTAGCCCATGCAGGAGCTTCAGT
AGATCTAACCATTTTCTCTTTACACTTAGCAGGAGTTTCCTCAATTTTAGGAGCCATCAACT
TCATTACAACAATTATCAACATAAAGCCCCCCGCAATGTCACAATACCAAACCCCTCTGTTC
GTATGATCCGTAATAATTACCGCCGTACTACTACTACTCTCGCTCCCTGTATTAGCAGCCGG
CATCACAATGCTATTAACAGACCGGAACCTAAATACAACCTCNTTCCGAACCGGAA
```

Supplementary Fig. S3 *Cytochrome c oxidase subunit I* (*COI*) gene of the cultured bovine foetal fibroblasts, sequenced by Sanger sequencing.

```
>LEUTX
ATGGCAGAAAAACAAAATTCAACTCGGCGATTCCGCACACGCTTTAATGGAGAACAGCTAGG
AGCACTAAGAGATGTATTTGAAAGGACCAGGTACCCACATTGTTTCCTCATAAGAACACTTG
CTTCAACTATTCATCTTGATGCGTCAGTTATAAAGACTTGGTTTAAAAACCAACGTGTCAAA
AGGAGGAGGGAGGAGAATCAGACTCAGCAAAATCTGTCACCAGGAGACCCGCGCCGGGTTGT
CTCAGTGAAGGAGGAAGAGATGCCCTTACCGGGCACTTCCGGAAGCACTCATCCCACGAGTC
TCAGCCTTGCAGGTGATTCTCATCACGAGCTACCTGAGCTTTCTTGTGCTGAGCAGTGTGAA
GGGGCTGCTGCCACTCCATGCCCTTCATCCTGCAATTTCCTGACTGCTCTAAGTCTCAGAGA
CACTGATCTTCCTTGGGCCTCCAGTCCTTATGACATGGATCAACTTATACAGTTATACGACT
TACCTGGGGATGATGACCCCAGCAGTCTGGATCAGTACCTCTTCCCAGAGTGCTCCAGCTGG
GGGACTGTGGCCGGCACTGATCACCATCATGAGGACGAACACAGCTACAATCCTGAGAAAGT
TCCGGGCGGGGGGGGCTCCGGAGGAGGTGGCAGCGACTACAAAGACCATGACGGTGATTATA
AAGATCATGACATCGATTACAAGGATGACGATGACAAGTGA

>ARGFX
ATGGGCACTCCAAGGGAATTCATGACAGATTTCCCTTGCCAGACATCAGCACCCCTGATGAA
CAAAAGAGTCCAAGAGAATCCCCTGCCAGACCCTTTTGTCTACATGGATGATTCTAGCACGA
ACTTCCAGGATGATTCAAAGATACCAGCTGGTGCCAGTCTTTTTTCATCACGGCCTACGACG
TGTGGAATCTTAACTCCCCAACCAGGGATCAAACCCGGGCCTTCGACAGTGAAAGCAAAATG
GAAGAAGAGTCATGAACGCACCTCATTCACCCACACACAGTACAAGGAGTTGGAGGCTCTGT
TTAGCTGCAACATGTTTCCAGATAAAAACCTCCAGAGAGAACTTGCTTTAAAACTCAATCTA
CCAGAGTCAACAGTAAAGATTTGGTTCAGGAACCGGCGGTTCAAAATGAAGAAGCAGCAGCG
GGAGCAAGAGCAGCAATCACTAAAGCCACCAAGCCAGGTCCTTCCAGCCAAGGATGTGCCCA
CAGTATCAACCAGCCCTCATTCTTTTCTCCTTGCAATTTCAGATTCCTATAACTCCCTCTCA
CCTCAGCCCTTAGACACTTTCCCCTGGGCAGGGGACTCTATGATCATGGAGATTCCTACAAG
TGATGTCCAAACGCAAGATCCTCAACTGGAGAGGCTAGTGGCCTCAGTTCCTGCTTTGTACT
CTGATGCATTTGACATCACCCAAATCATGGAACTGTACAGTGTTCCTGATGAGGATGACATC
ACCAACTCTTCCTTCTATTCTCTATATCAGTATCTCTCACCGACAAGGCCCAGTGGCGGGGG
GGGCTCCGGAGGAGGTGGCAGCGACTACAAAGACCATGACGGTGATTATAAAGATCATGACA
TCGATTACAAGGATGACGATGACAAGTGA

>ARGFX mutant
ATGGGCACTCCAAGGGAATTCATGACAGATTTCCCTTGCCAGACATCAGCACCCCTGATGAA
CAAAAGAGTCCAAGAGAATCCCCTGCCAGACCCTATGATTCTAGCACGAACTTCCAGGATGA
TTCAAAGATACCAGCTGGTGCCAGTCTTTTTTCATCACGGCCTACGACGTGTGGAATCTGGC
GGGGGGGGCTCCGGAGGAGGTGGCAGCGACTACAAAGACCATGACGGTGATTATAAAGATCA
TGACATCGATTACAAGGATGACGATGACAAGTGA
```

Supplementary Fig. S4 Sequences of ectopically expressed ETCHbox genes, including GGGGSGGGGS linkers (blue) and 3xFLAG (red).

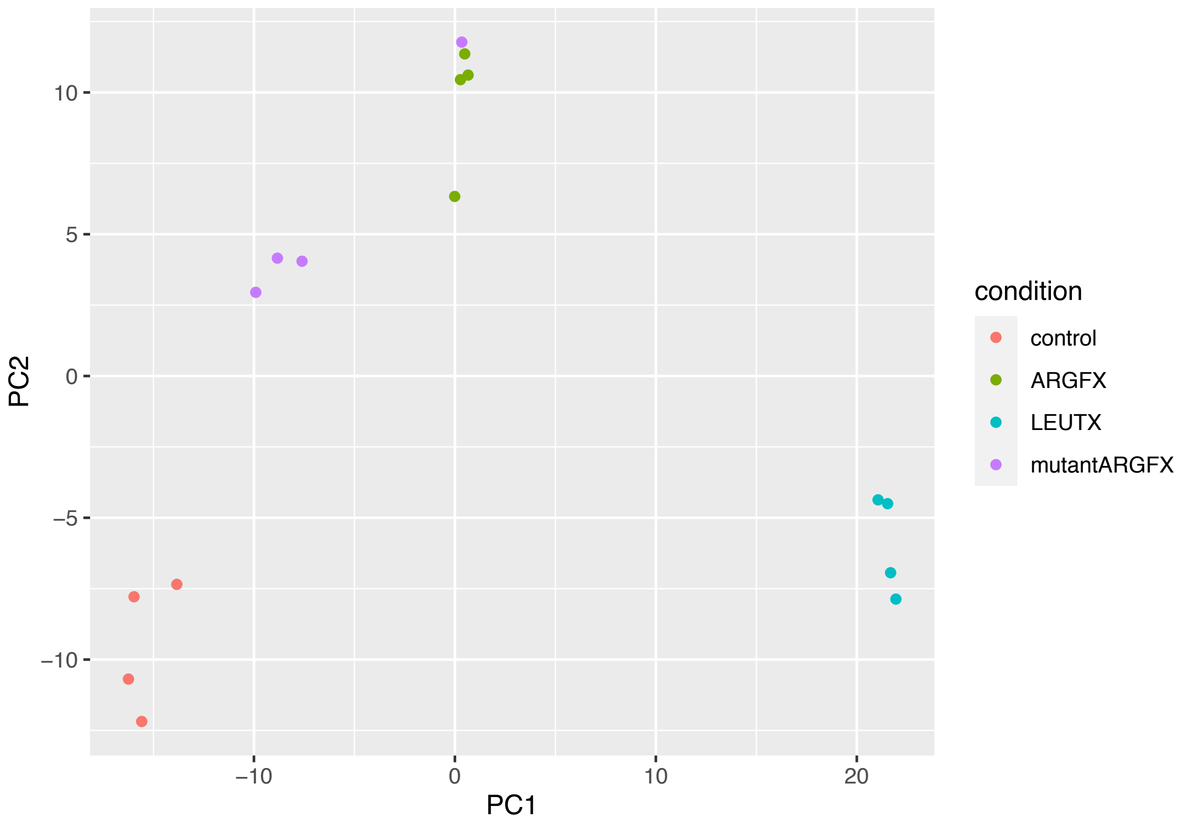

Supplementary Fig. S5 Principle Component Analysis (PCA) to test for outliers in the RNA-seq replicates. Figure shows PCA biplot including control, *LEUTX*, WT *ARGFX* and mutant *ARGFX* samples. *LEUTX*, WT *ARGFX* and mutant *ARGFX* samples are well separated by PCA, except for one *ARGFX* mutant sample, which is an outlier, clustering with WT *ARGFX*. This sample was removed before differential expression analysis.
